# Orobol, an Enzyme-Convertible Product of Genistein, exerts Anti-Obesity Effects by Targeting Casein Kinase 1 Epsilon

**DOI:** 10.1038/s41598-019-43950-9

**Published:** 2019-06-20

**Authors:** Hee Yang, Sang‐Hyuk Lee, Hae Ji, Jong-Eun Kim, Ra Yoo, Jong Hun Kim, Sujin Suk, Chul Sung Huh, Jung Han Yoon Park, Yong-Seok Heo, Han-Seoung Shin, Byung-Gee Kim, Ki Won Lee

**Affiliations:** 1 Department of Agricultural Biotechnology and Research Institute of Agriculture and Life Sciences, Seoul National University, 08826 Seoul, Republic of Korea; 2 Advanced Institutes of Convergence Technology, Seoul National University, 16229 Suwon, Republic of Korea; 3 Interdisciplinary Program for Biochemical Engineering and Biotechnology, Seoul National University, 08826 Seoul, Republic of Korea; 4 Research Institute of Biotechnology and Medical Converged Science, Dongguk University, 10326 Goyang, Republic of Korea; 5 Department of Food Science and Biotechnology, Sungshin University, 01133 Seoul, Republic of Korea; 6 Graduate School of International Agricultural Technology, Seoul National University, 25354 Pyeongchang, Gangwon-do Republic of Korea; 7 Department of Chemistry, Konkuk University, Seoul, 05030 Republic of Korea; 8 Department of Food Science and Biotechnology and Food and Biosafety Research Center, Dongguk University-Seoul, Ilsandong-gu, 10326 Gyeonggi-do, Republic of Korea; 9 School of Chemical and Biological Engineering, Seoul National University, 08826 Seoul, Republic of Korea; 10 BOBSNU Co., Ltd, 16229 Suwon, Gyeonggi-do Republic of Korea

**Keywords:** Target identification, Molecular medicine

## Abstract

Soy isoflavones, particularly genistein, have been shown to exhibit anti-obesity effects. When compared with the isoflavones genistin, daidzin, coumestrol, genistein, daidzein, 6-o-dihydroxyisoflavone, equol, 3′-o-dihydroxyisoflavone, and 8-o-dihydroxyisoflavone, a remarkably higher inhibitory effect on lipid accumulation was observed for orobol treatment during adipogenesis in 3T3-L1 cells. To identify the cellular target of orobol, its pharmacological effect on 395 human kinases was analyzed. Of the 395 kinases, orobol showed the lowest half maximal inhibitory concentration (IC_50_) for Casein Kinase 1 epsilon (CK1ε), and bound to this target in an ATP-competitive manner. A computer modeling study revealed that orobol may potentially dock with the ATP-binding site of CK1ε via several hydrogen bonds and van der Waals interactions. The phosphorylation of eukaryotic translation initiation factor 4E-binding protein 1, a substrate of CK1ε, was inhibited by orobol in isobutylmethylxanthine, dexamethasone and insulin (MDI)-induced 3T3-L1 cells. It was also found that orobol attenuates high fat diet-induced weight gain and lipid accumulation without affecting food intake in C57BL/6J mice. These findings underline orobol’s potential for development as a novel agent for the prevention and treatment of obesity.

## Introduction

Obesity is defined as an excessive accumulation of fat that is sufficient to adversely affect health^1–3^. The growing prevalence of obesity and overweight has become a worldwide health problem with significant social costs^1–3^. During conditions of energy surplus, adipose tissue enlarges through a combination of hypertrophy (increase in cell size) and hyperplasia (increase in cell number). Adipocytes are fat cells and critical regulators of whole-body metabolism that are created from precursor cells^4^. Both hypertrophy and hyperplasia of adipocytes are considered to be crucial targets for the prevention and treatment of obesity. Adipogenesis is a multi-step process leading to adipocyte development^5,6^, with adipocyte differentiation involving a temporally regulated set of gene-expression events involving the nuclear receptor peroxisome proliferator activated receptor γ (PPARγ) and CCAAT/enhancer-binding protein α (C/EBPα). Adipogenesis consists of an early stage of proliferation and terminal differentiation^7^.

Kinases play key roles in signal transduction for growth factors, hormones, cytokines and neurotransmitters to translate intracellular signals^8,9^. These signals can lead to alterations in gene expression, cell division, cell death and metabolism and play roles in almost all diseases related to dysfunctional signaling pathways^10^. Therefore, kinases represent excellent drug targets as signal transduction components^8^. Iressa and Gleevec are good examples of drugs that target kinases^11^. Numerous studies have shown how the signaling pathways of kinases play roles in adipogenesis^1^.

Casein kinase 1 (CK1) is the key kinase that regulates circadian rhythm. Circadian rhythm governs a remarkable range of metabolic and physiological functions^12–14^. The CK1 family phosphorylates crucial regulatory processes including cell proliferation and differentiation^15^. CK1ε is a regulator of eukaryotic translation initiation factor 4E-binding protein 1 (4E-BP1) phosphorylation, a regulator of adipogenesis and metabolism in mammals^16–20^. 4E-BP1 also plays a critical role in controlling biological processes, including cell proliferation and protein synthesis^21^. The importance of 4E-BP1 is that it inhibits the initiation of mRNA translation by binding to eukaryotic translation initiation factor 4E (eIF4E)^22^. 4E-BP1 phosphorylation results in eIF4E release, thereby enabling cap-translation initiation^23^.

Currently available anti-obesity drugs are associated with numerous side-effects, including dry mouth, high blood pressure, constipation, headache, and insomnia. For this reason, natural compounds have been the focus of alternative strategies for combating the disease^24^. In some Asian countries, a typical diet consists of high levels of soy and soy-based products. The lower frequency of obesity and related metabolic diseases in these countries has been associated with soy consumption, with particular regard to isoflavone content^25^. Orobol is a rare isoflavone derived from soybean that exists in very minute amounts in nature and can arise during the fermentation of soybeans or metabolism in the body^26,27^. Orobol is structurally similar to genistein which is the most prevalent soy isoflavone. Genistein is hydroxylated to form orobol in the body and orobol formation is reportedly essential for its anti-obesity activity^28^. Orobol has been reported to inhibit angiogenesis and the proliferation of endothelial and cancer cells^27,29,30^. Because orobol is a rare isoflavone, we have developed an efficient enzyme-based method for orobol production from genistein^31^. Orobol may now potentially be developed into a food supplement or medicine with greater economic rationale. In the present study, we sought to investigate the anti-obesity effect and the mechanisms of orobol in 3T3-L1 preadipocytes.

## Results

### Orobol exhibits the most potent inhibitory effects among soy isoflavones on isobutylmethylxanthine, dexamethasone and insulin (MDI)-induced adipogenesis in 3T3-L1 preadipocytes

**Figure 1:**
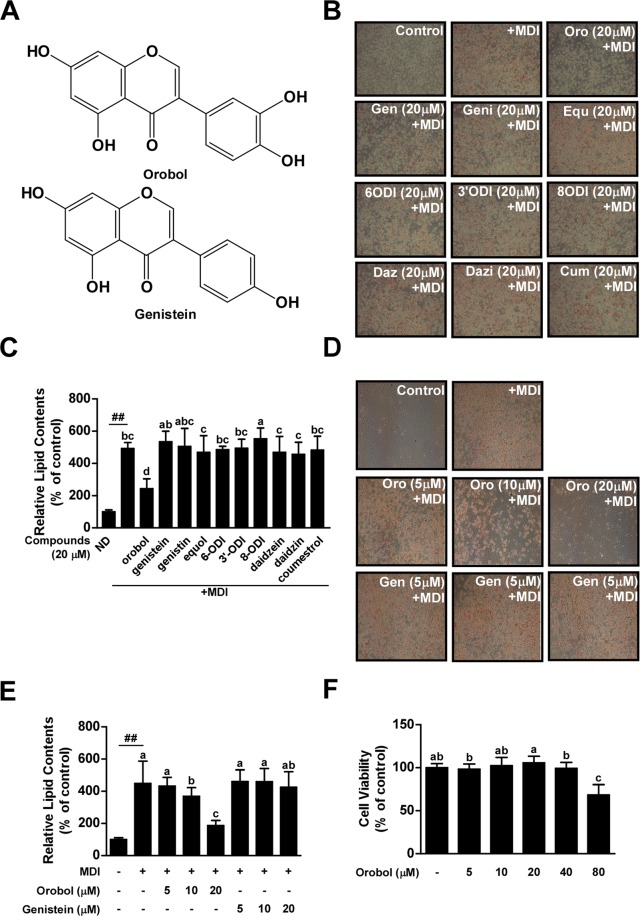
Effects of orobol on MDI-induced adipogenesis in 3T3-L1 preadipocytes. (**A**) The chemical structures of orobol and genistein. (**B**,**C**) Orobol is the most effective inhibitor of 3T3-L1 preadipocyte differentiation among the tested soy isoflavones and their metabolites. (**B**) After cell differentiation, 3T3-L1 adipocytes were stained with Oil red O and images were captured. (**C**) Quantification of intracellular lipid content. (**D**,**E**) Orobol, but not genistein, inhibits MDI-induced adipogenesis in 3T3-L1 cells. (**F**) Orobol does not exhibit cytotoxicity in 3T3-L1 preadipocytes up to 40 μM. Data are represented as means ± SEM from at least three independent experiments. The sharps (##) indicate a significant difference between the control group and the group treated with the MDI cocktail alone (*p* < 0.01). Means with different letters (a–d) within a graph are significantly different between groups treated with MDI alone and those treated with MDI plus orobol or other soy isoflavones.

To examine the anti-adipogenic effect of the soy isoflavones genistin, daidzin, coumestrol, genistein, daidzein, and their metabolites orobol, 6-o-dihydroxyisoflavone (6-ODI), equol, 3′-o-dihydroxyisoflavone (3′-ODI), and 8-o-dihydroxyisoflavone (8-ODI), 3T3-L1 preadipocytes were treated with MDI and each compound simultaneously at 20 μM. MDI significantly increased the relative lipid contents by 5-fold compared to the undifferentiated control. Oil Red O (ORO) staining indicated that orobol inhibited adipocyte differentiation at 20 μM, whereas the other soy isoflavones and their metabolites had no detectable effect (Fig. 1B,C). To compare the anti-adipogenic effect between orobol and its precursor, genistein, ORO staining was conducted in cells treated with various concentrations of orobol or genistein. MDI-induced lipid accumulation was reduced in the cells treated with 10 or 20 μM orobol, whereas the same concentrations of genistein had no effect (Fig. 1D,E). To determine whether the decreased lipid accumulation by orobol was attributable to diminished cell viability, an MTT assay was performed. Orobol at 5~40 μM concentrations did not decrease cell viability (Fig. 1F).

### Orobol blocks MDI-induced lipid accumulation through all stages of adipogenesis in 3T3-L1 preadipocytes

**Figure 2:**
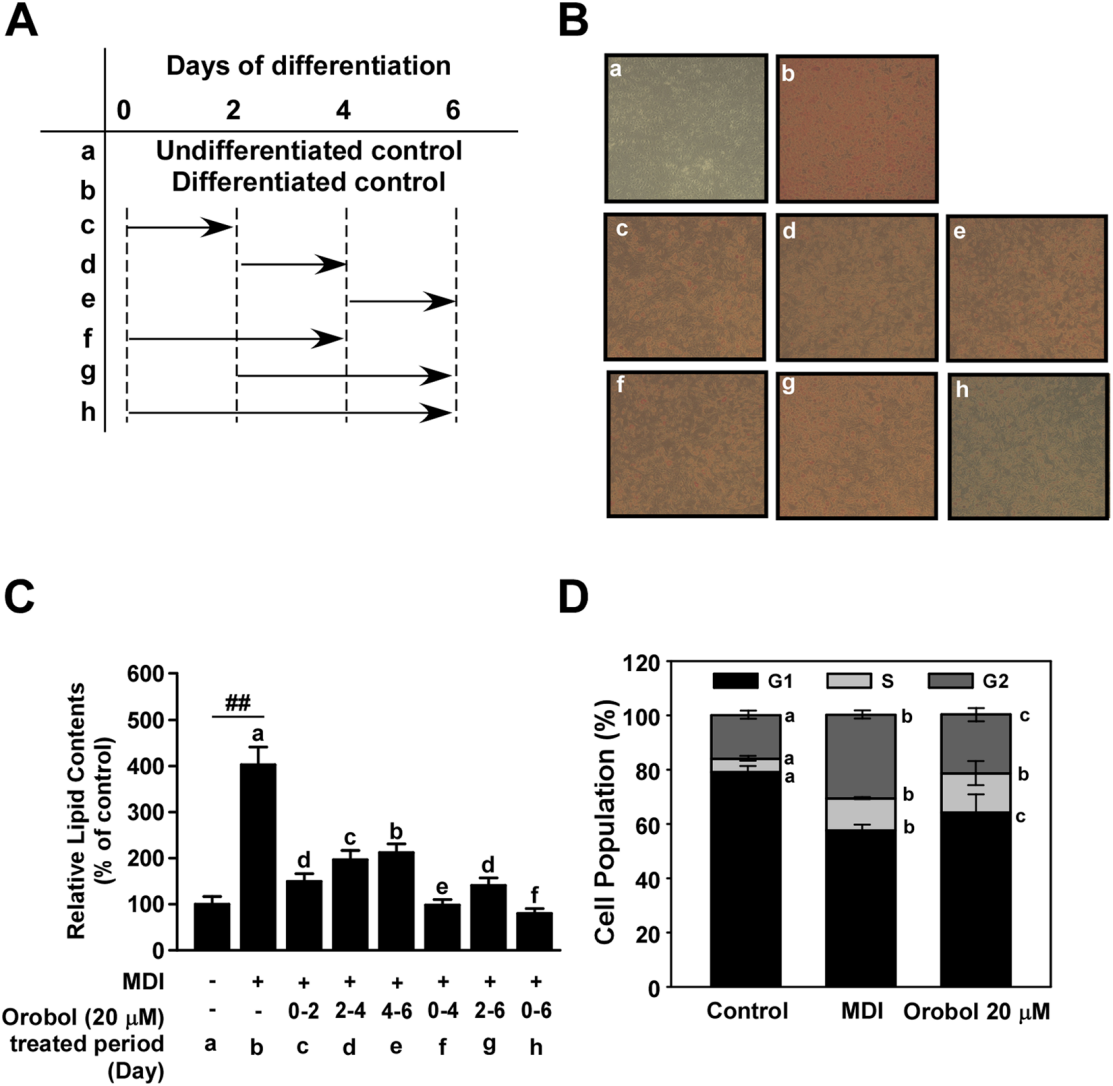
Effects of orobol on 3T3-L1 preadipocytes at different stages of MDI-induced adipogenesis. (**A**) A time schedule for orobol treatment during cellular differentiation. (**B**,**C**) Confluent 3T3-L1 preadipocytes were differentiated into mature adipocytes in the presence of 20 μM orobol for the indicated time periods. (**B**) After differentiation, the images of 3T3-L1 adipocytes stained with Oil Red O solution were captured. (**C**) Stained lipids were quantified via spectrophotometry as described in the Materials and Methods. The sharps (##) indicate a significant difference between the control group and the group treated with MDI alone (*p* < 0.01). Means with different letters (a–f) within a graph are significantly different between groups treated with MDI alone and those treated with MDI plus orobol treated at each of the different stages. (**D**) Orobol delays MDI-stimulated cell cycle progression at the G1 phase. When 3T3‐L1L preadipocytes reached confluence, orobol (20 μM) or daidzein in DMEM supplemented with 10% FBS containing MDI was added, and the extent of cell proliferation examined. After the indicated time periods (16 h), cells were dissociated with trypsin/EDTA and FACS analysis was conducted. Data are representative of three independent experiments that yielded similar results. Data are represented as mean ± SEM from at least three independent experiments. Means with different letters (a–c) within a graph are significantly different between groups.

Adipogenesis consists of early, intermediate, and terminal phases of differentiation. To identify the key stage at which orobol exerts its anti-adipogenic activity, orobol was treated at different stages of cellular differentiation (Fig. 2A). Adipogenesis overall was reduced regardless of when orobol was added: however, the degree of inhibition was the highest when orobol was present between 0–2 days, followed by 2–4 days and <4–6 days (Fig. 2B,C). In early stage of adipogenesis (0–2 days), proliferation of preadipocytes is a main event^7^. Previous studies have demonstrated that orobol inhibits cell proliferation in endothelial cell or breast epithelial cells each exerting anti-angiogenic or anti-cancer effects^32,33^. Cell cycle progression was measured using FACS analysis and the population of cells in each cell cycle phase was quantified. Control cells were predominantly within G1 phase (Fig. 2D and Supplement Fig. 1). MDI treatment stimulated cell cycle progression, evidenced by a greater proportion of total cells entering S phase at 16 h treatment. Interestingly, the majority of the cells was arrested in G1 phase after 16 h of treatment with orobol. Collectively, these results indicate that orobol suppresses MDI-induced cell proliferation of 3T3-L1 preadipocytes by retarding cell cycle progression, which is consistent with the inhibitory effect of orobol on the proliferation of other cell types.

### Orobol inhibits CK1ε kinase activity

**Figure 3:**
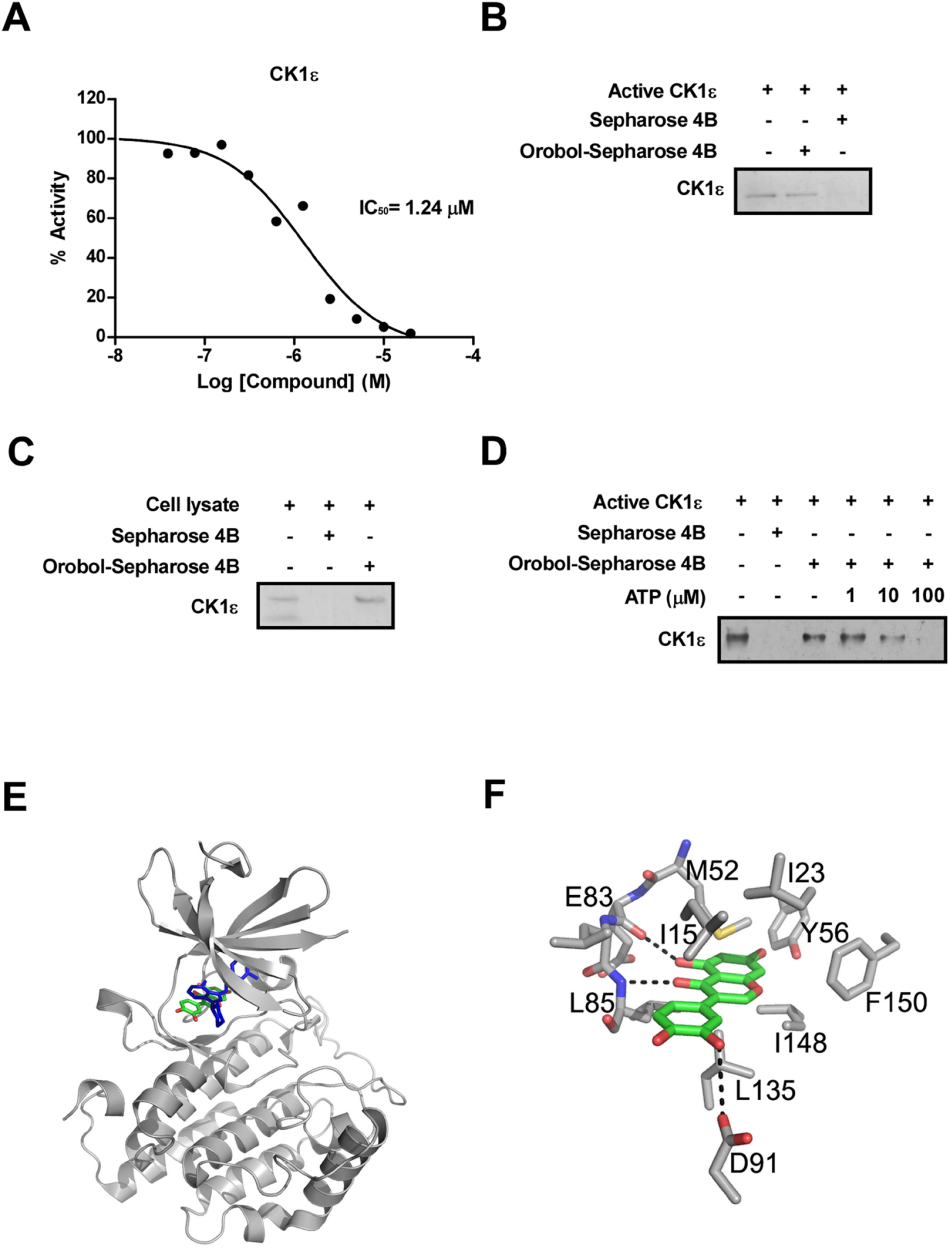
Inhibitory effects of orobol on CK1**ε** kinase activity. (**A**) Orobol was tested for CK1**ε** inhibitory activity in ten concentrations with 2-fold serial dilutions starting at 20 μM. (**B**) Orobol binds to CK1**ε** directly *in vitro*. The orobol binding was evaluated by immunoblotting using an antibody against CK1**ε**: lane 1, CK1**ε**; lane 2, CK1**ε** kinase bound to orobol-Sepharose 4B beads and lane 3, CK1**ε** precipitated with Sepharose 4B. (**C**) Orobol directly interacts with CK1**ε** in 3T3-L1 cell lysates. The CK1**ε** kinase bound to orobol was evaluated by immunoblotting: lane 1, CK1**ε** kinase in whole lysates of 3T3-L1 cells; lane 2, CK1**ε** kinase in lysates precipitated with Sepharose 4B beads; and lane 3, CK1**ε** in whole lysates of 3T3-L1 cells precipitated by orobol-Sepharose 4B beads. (**D**) Orobol binds to CK1**ε** in an ATP-competitive manner. CK1**ε** (0.2 µg) was incubated with ATP at the indicated concentrations (0, 10, or 100 μM) together with 100 µl orobol-Sepharose 4B beads or Sepharose 4B beads (negative control) added in reaction buffer to a final volume of 500 µl. The immunoprecipitated proteins were detected by immunoblotting with an antibody against CK1**ε**. Lane 2, negative control, showing that CK1**ε** does not bind to Sepharose 4B beads alone; lane 3: positive control, showing that CK1**ε** binds with orobol-Sepharose 4B beads. Presented signals from were cropped from one continuous Western blot which is displayed as Suppl. Figure (**E**,**F**) Model structure of CK1ε in complex with orobol (**E**) and the detailed interaction of the complex (**F**). Orobol (atomic color) binds to the ATP-binding site of CK1ε; PF4800567 (blue) is overlaid for comparison. The residues involved in the interaction with orobol are labeled and the hydrogen bonds are depicted as dotted lines.

Kinase profiling analysis was conducted to identify kinases that were inhibited by orobol. First, 395 human kinases were examined with 20 μM orobol (Supplement Table 1). Of these 395 kinases, the activity of 26 kinases was found to be inhibited completely. Kinase profiling analysis was repeated with these 26 kinases at 1 μM orobol (Table 1). Based on the results presented in Table 1, We selected MUSK, TNIK, MNK1, KHS/MAP4K5, TOPK and CK1**ε** whose activities were inhibited more than 75% by orobol and measured the half maximal inhibitory concentration (IC_50_) of orobol for these kinases (Table 2). In previous studies, isoflavonoids including orobol inhibited the activity of phosphoinositide 3-kinase (PI3K)^34,35^. We also measured the IC_50_ of orobol in isoforms of PI3K (Table 3). Among these kinases candidates, CK1**ε** had the lowest IC_50_ of orobol, and orobol effectively attenuated the activity of the CK1**ε** kinase in a dose-dependent manner (Fig. 3A). To examine whether the orobol-mediated reductions in CK1**ε** kinase activity occurs through a direct interaction between orobol and CK1**ε** kinase, a pull-down assay was conducted. The CK1**ε** kinase bound to orobol–sepharose 4B beads, but not to control sepharose 4B beads (Fig. 3B), with orobol was co-precipitating with CK1**ε** in cell lysates (Fig. 3C). Next, to examine the mode of orobol binding to CK1**ε**, we performed ATP competitive-binding assays. ATP effectively inhibited orobol binding to CK1**ε** (Fig. 3D) suggesting that orobol binds with CK1**ε** in an ATP-competitive manner. To investigate the molecular basis for the ATP-competitive inhibition of CK1ε by orobol, a docking study was carried out using the crystal structure of CK1ε in complex with an ATP-competitive inhibitor, PF4800567, as a template model structure (Fig. 3E,F)^36^. We also found that PF-5006739, a potent and selective CK1ε inhibitor^37^, significantly inhibited MDI-induced adipogenesis at 0.625 ~ 5 μM without influencing cell viability (Supplement Fig. 2A–C).

**Table 1:** Kinase screening of orobol (1 μM).

Kinase	Activity	Kinase	Activity	Kinase	Activity	Kinase	Activity	Kinase	Activity
ACK1	91	CK1γ3	108	MLK2/MAP3K10	94	PBK/TOPK	74	TNIK	68
BTK	95	ERK2/MAPK1	110	MNK1	69	PLK2	80	ULK2	104
CAMK1α	98	HPK1/MAP4K1	103	MUSK	61	RON/MST1R	78		
CAMK1δ	90	IR	111	MYO3β	86	SGK2	98		
CDK5/p25	78	KDR/VEGFR2	76	NEK5	85	SRPK2	101		
CK1ε	74	KHS/MAP4K5	72	NEK6	79	TIE2/TEK	87		

26 kinases were selected using the criteria of inhibiting more than 100% of the kinase activity, based on Table 1. Kinase profiling analysis was conducted with 1 μM orobol. Data are representative of two independent experiments that offered similar results.

**Table 2:** The half maximal inhibitory concentration (IC_50_) of orobol for various kinases.

Kinase	IC_50_ (μM)
CK1ε	1.24
KDR/VEGFR2	4.45
KHS/MAP4K5	1.42
MNK1	2.13
MUSK	1.48
PBK/TOPK	1.67
TNIK	1.54

IC_50_ values were measured as described in the Materials and Methods.

**Table 3:** The IC_50_ of orobol for Phosphoinositide 3-kinase (PI3K) isoforms.

Kinase	IC_50_ (μM)
PI3Kα	3.46
PI3Kβ	5.44
PI3Kγ	4.76
PI3K (p110α/p65α)	3.65
PI3Kδ	5.27

IC_50_ values were measured as described in the Materials and Methods.

### Orobol suppresses 4E-BP1 phosphorylation in 3T3-L1 preadipocytes

**Figure 4:**
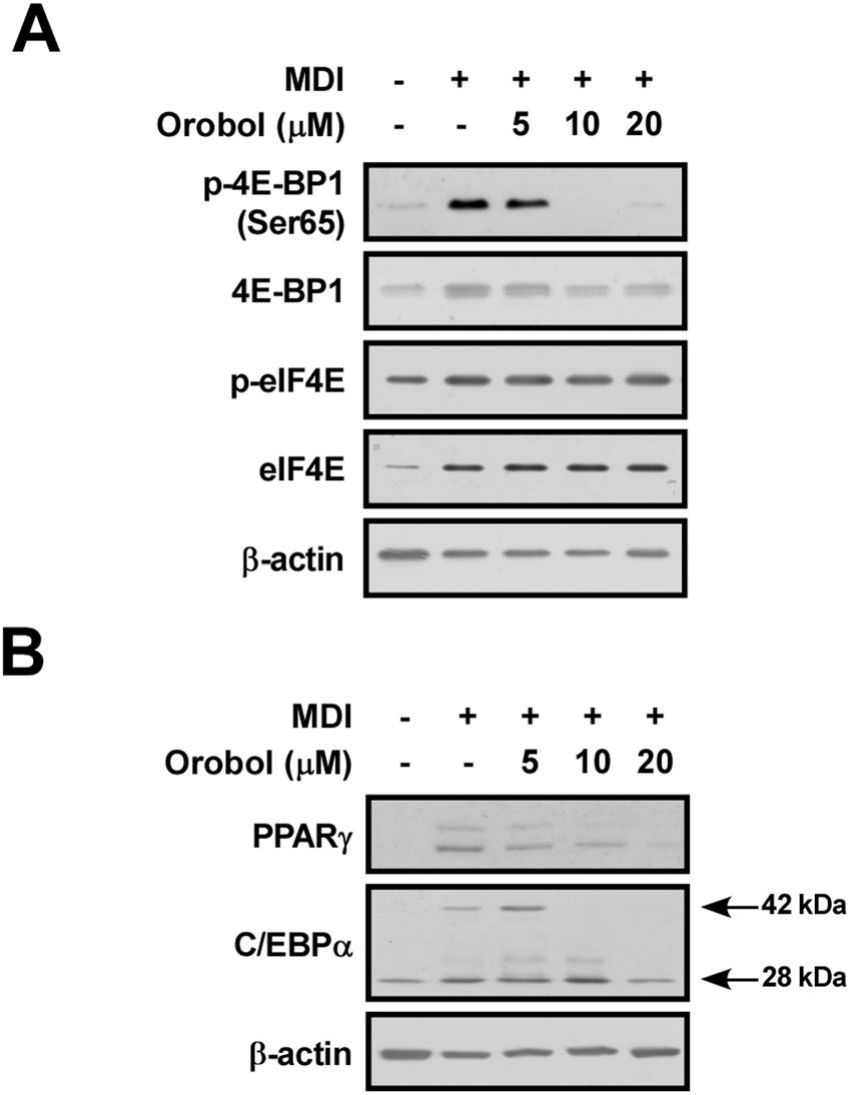
Effects of orobol on MDI-induced 4E-BP1 signaling in 3T3-L1 preadipocytes. (**A**) The protein expression levels of phospho- and total-4E-BP1, but not phospho- and total-eIF4E proteins, were downregulated by orobol dose-dependently. (**B**) Orobol suppressed PPARγ and C/EBPα expression in 3T3-L1 preadipocytes. Arrows marked on the band for C/EBPα point to specific C/EBPα proteins. The data are representative of three independent experiments that gave similar results. Presented signals from were cropped from one continuous Western blot which is displayed as Suppl. Figure.

To examine whether CK1ε activity is inhibited by orobol in the cell, we measured the phosphorylation of 4E-BP1 as a known substrate of CK1**ε**^38^. 4E-BP1 has been shown to negatively regulate eIF4E^39^, and orobol effectively suppressed MDI-induced phosphorylation of 4E-BP1 (Fig. 4A). PPARγ and C/EBPα are master regulators of adipogenesis^40^. The protein expression levels of these proteins were also attenuated by 10–20 μM of orobol in 3T3-L1 preadipocytes treated with MDI (Fig. 4B). Collectively, these findings indicate that orobol inhibits 4E-BP1-mediated adipogenic signaling pathways stimulated by MDI.

### Orobol mitigates HFD-induced weight gain in mice

**Figure 5:**
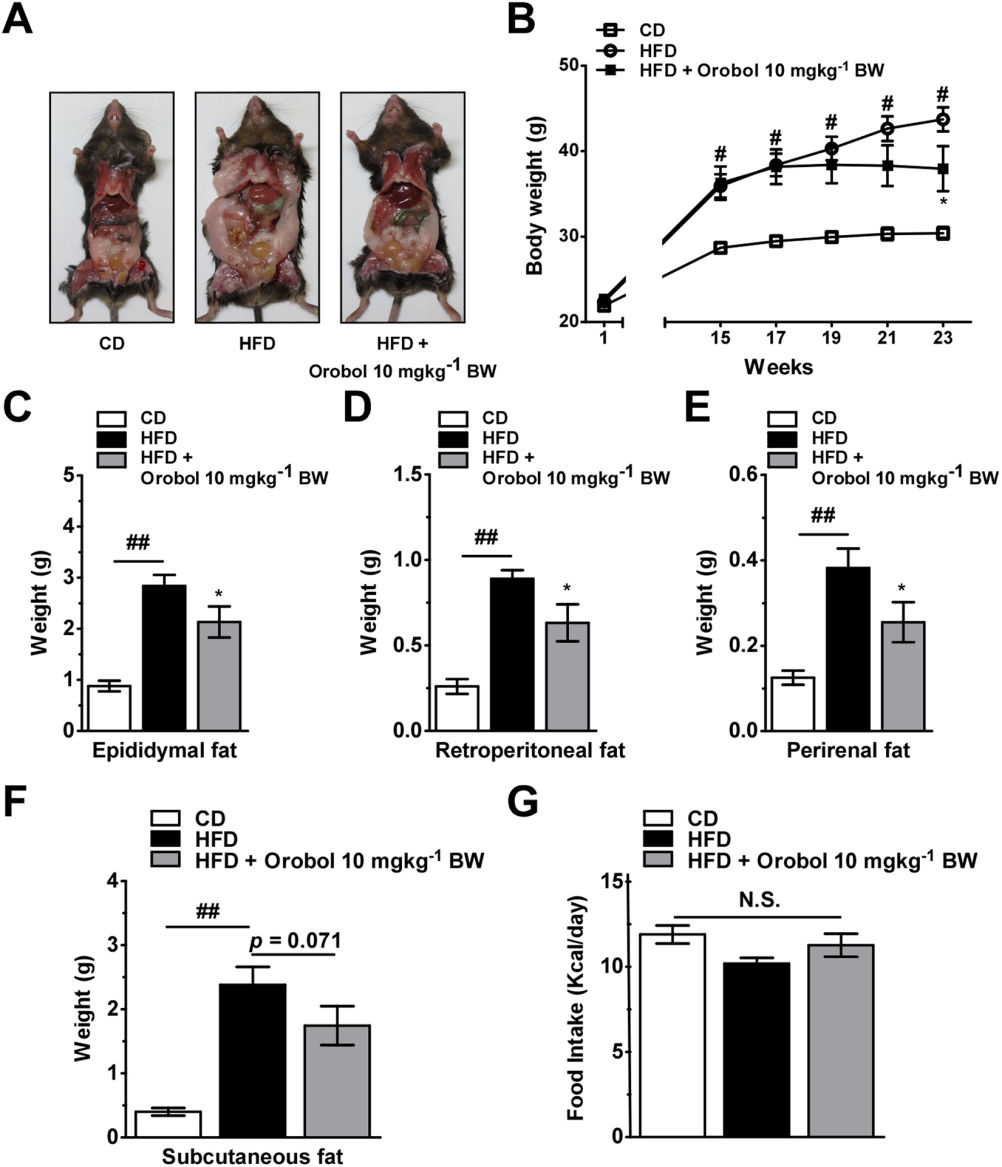
Effects of orobol on HFD-induced obesity in C57BL/6J mice (**A**) The photographs of the C57BL/6J mice at autopsy showed fat mass reduced by orobol treatment. (**B**) Orobol treatment for 23 weeks alleviated HFD-induced weight gain in C57BL/6 J mice. (**C**–**E**) Oorobol decreased epididymal fat, retroperitoneal fat, and perirenal fat masses. (**F**) Orobol has no significant effect on subcutaneous fat mass. (**G**) Caloric intake was unaffected by orobol (10 mgkg^−1^ BW). Values are expressed as means ± SEM. The sharps (# or ##) indicate a significant difference between the control group and the group treated with HFD (*p* < 0.05 or *p* < 0.01). The means marked with ‘*’ indicate significantly different compared to the group treated with HFD (p < 0.05).

To further investigate the anti-obesity effects of orobol, mice were fed HFD in the presence or absence of orobol (10 mg/kg^−1^ BW) for 23 weeks. Photographic data showed that orobol supplementation resulted in a less obese phenotype, which might be associated with decreased fat accumulation (Fig. 5A). The average body weight of the HFD-fed mice (43.72 ± 1.41 g) was approximately 30.5% higher than that of the control mice (30.40 ± 0.72 g). Administration of orobol 10 mg/kg^−1^ BW significantly reduced body weight by 17.3% compared to the HFD group (p < 0.05; Fig. 5B). The autopsy results indicated that orobol significantly reduced visceral fat mass including epididymal, retroperitoneal, and perirenal fat in the HFD-fed mice (p < 0.05; Fig. 5C–E). Additionally, orobol administration tended to decrease subcutaneous fat mass in the HFD-fed mice (p = 0.071; Fig. 5F). There were no significant differences (p > 0.05) in daily caloric intake (kcal/day) between the HFD and orobol 10 mg kg^−1^ BW groups (Fig. 5G).

## Discussion

Soybean has been used as traditional protein source for centuries in Asia^41^. The legume is a rich source of vitamins and minerals and a complete protein source rich in all of the essential amino acids^42^. Recently, the numerous beneficial effects of soybean on human health have been the focus of research, including preventive effects against cancer and metabolic diseases^43^. Isoflavones are bioactive components of soybean^44^ and act as estrogen and kinase inhibitors^45^. Genistein, daidzein and equol are among the most well-known soy isoflavones, with rarer isoflavones generated during metabolism and the fermentation process^44^. Orobol is rare in nature and found only in trace amounts in fermented foods^26,28,29,32,46^. Recently, it has been reported that the soy isoflavone metabolite 6,7,4′-trihydroxyisoflavone exerts anti-adipogenic effects that are more potent than its precursor, daidzein^47^. However, there is a paucity of studies on the anti-obesity effects of orobol.

In the present study, we newly demonstrated that orobol effectively inhibited adipocyte differentiation compared to its precursor, genistein. Consistent with the anti-adipogenic effect of orobol, PF-5006739, a potent and selective CK1ε inhibitor, treatment significantly inhibited adipogenesis. CK1ε plays essential roles in diverse cellular processes including transcription and translation processes responsible for generating circadian rhythm in mammals^48,49^, and it serves as the key regulator of circadian timing^50^. Previous studies have shown that disruption of the circadian clock has been linked to obesity and metabolic diseases^12,51,52^. Cunningham P.S *et al*. (2016) have demonstrated that daily administration of PF-5006739 improves glucose homeostasis both in HFD-induced and genetic-induced obese mice^37^. The evidences from these studies support that orobol could be a novel anti-obesity agent as a natural CK1ε inhibitor achieving metabolic benefits in obesity.

Computer modelling suggests that orobol might dock to the ATP-binding site of CK1ε through the formation of several hydrogen bonds and van der Waals interactions. The compound may form hydrogen bonds with the backbone carbonyl and amide groups of Glu83 and Leu85 and the side chain of Asp91. In such an orientation, the inhibitor would be surrounded by the side chains of the hydrophobic residues in the ATP-binding site, including Ile15, Ile23, Met52, Tyr56, Leu135, Ile148, and Phe150. The highly inhibitory activity of orobol for CK1ε would be due to these hydrogen bonds and hydrophobic interactions. Further studies with X-ray crystallography to determine the complex structure will elucidate its exact binding mode to CK1ε.

4E-BP1 is a substrate of CK1ε that modulates cell proliferation and differentiation^16,21–23^. Hyperphosphorylation of 4E-BP1 results in a loss of binding ability with eIF4E and a subsequent loss of translational activity^53^. 4E-BP1 influences PPARγ and C/EBPα activity, which are master regulators of adipogenesis^54,55^. We observed that the phosphorylation of 4E-BP1 was reduced and consequently, the expression of the key regulators of adipogenesis including PPARγ, C/EBPα induced during adipogenesis in 3T3-L1 preadipocytes as well as cell cycle progression were inhibited by orobol treatment. On the other hands, orobol treatment had no effect on the expression of Akt/mTORC1 signaling proteins as a well-known upstream signaling of adipogenesis (Ref)(Supplement Fig. 3). These results support that the anti-obesity effect of orobol was attributed to activity of CK1ε but not on other targets such as PI3K. Mechanistically it is suggested that dephosphorylation of 4E-BP1 is secondary to proliferation and differentiation of preadipocytes rather than inhibition of CK1ε.

Our data shed light on important aspects of anti-obesity effect of orobol. Since adipose tissue is a major organ to contribute to increase of body weight^56,57^, we mainly focused on the effect of orobol on adipocytes and adipose tissue in this study. However, it is possible that the effect of orobol may not be limited to the adipocytes and adipose tissues. We observed that the weight of liver tissue were decreased in HFD + orobol-fed mice compared to HFD-fed mice (Supplement Fig. 4). This result implies that orobol may also play a role in non-adipose tissue including liver and skeletal muscle. Although we did not explore the expending effects of orobol on other non-adipose tissues, it is required to further study about potential effect of orobol on other aspects such as locomotor activity.

In summary, this study provides the first evidence that orobol inhibits adipogenesis in 3T3-L1 adipocytes by reducing CK1ε kinase activity via direct binding. Orobol also exhibits anti-obesity effects in diet-induced obese mice, which is attributable to decreased adipose tissue mass. Taken together, these findings underline the potential for orobol to be developed for the prevention of obesity in a tissue-specific manner.

## Methods

### Reagents

Orobol was produced as described in our previous study^31^ via the bioconversion of genistein using tyrosinase which leads to ∂-hydroxylation^42,58^ in *Bacillus megaterium*. Polyethylene glycol 200 (PEG 200), 3-isobutyl-1-methylcanthine (IBMX), dexamethasone (DEX), human insulin, genistin, genistein, daidzin, daidzein, and equol were purchased from Sigma-Aldrich (St. Louis, MO). 6,7,4′-Trihydroxyisoflavone (6-ODI) was obtained from Chromadex™ (Irvine, CA). 7,3′,4′-Trihydroxyisoflavone (3′-ODI) and 7,8,4′-trihydroxyisoflavone (8-ODI) were purchased from Indofine Chemical (Hillsborough, NJ). 3-[4,5-Diethylthiazol-2-yl]-2,5-diphenyltetrazolium bromide (MTT) was purchased from USB (Cleveland, OH). CK1ε and eIF4E antibodies were purchased from Santa Cruz Biotechnology (Dallas, TX). Antibodies against phosphorylated 4E-BP1 and 4E-BP1 were purchased from Cell Signaling Biotechnology (Danvers, MA). The antibody against phosphorylated eIF4E was purchased from EPITOMICS (Burlingame, CA).

### Cell culture and preadipocyte differentiation

3T3-L1 preadipocytes (American Type Culture Collection, Manassas, VA) were cultured in Dulbecco’s modified eagle medium (DMEM) supplemented with 10% bovine calf serum at 5% CO_2_ and 37 °C until 100% confluence. After post-confluence (day 0), cells were incubated in DMEM supplemented with 10% fetal bovine serum (FBS) and adipogenic cocktail (MDI) comprising a mixture of 0.5 mM IBMX, 1 μM dexamethasone (DEX) and 5 μg/mL insulin for 2 days in order to induce differentiation. After 2 days, medium was changed to DMEM containing 10% FBS and 5 μg/mL insulin. Two days later, the medium was switched to DMEM containing 10% FBS until the preadipocytes were fully differentiated.

### Cell viability assay

3T3-L1 cells were seeded in 24-well plates at a density of 5.0 × 10^4^ cells per well. After reaching confluence, the monolayers were treated with orobol at concentrations of 5 ~ 80 μM for 72 hours. MTT (0.5 mg/mL) was then added and the incubation was continued for 1 hour at 37 °C to allow the formation of violet crystals (formazan). The crystal form of formazan was dissolved in dimethylsulfoxide (DMSO), and the absorbance was measured at 595 nm with a microplate reader (Beckman-Coulter, CA).

### Oil Red O staining

3T3-L1 cells were seeded in 24-well plates at a density of 5.0 × 10^4^ cells per well. After reaching confluence, the cells were differentiated for 6 days in the presence or absence of the tested bioactive compounds. Differentiated cells were subjected to Oil Red O staining to visualize accumulated lipid droplets in the cells. The media was removed and differentiated cells were fixed with 4% formalin for 20 min, followed by phosphate buffered saline (PBS) washing. The fixed cells were then stained with Oil Red O (5 mg/L 60% isopropyl alcohol) for 15 min at room temperature. After staining, the cells were washed three times with PBS. Intracellular lipid content was quantified by eluting Oil Red O stain with isopropyl alcohol and quantifying at 515 nm with a spectrophotometer (Beckman-Coulter, CA).

### Western blot assay

After confluence, differentiation of 3T3-L1 preadipocytes was induced in the presence or absence of the indicated concentrations of orobol as described above. Cell lysates were prepared and the protein concentration of each sample was determined. The protein concentration was measured using a dye-binding protein assay kit as described by the manufacturer (Bio-Rad Laboratories, Hercules, CA). Proteins in cell lysates were separated on sodium dodecyl sulfate polyacrylamide gel electrophoresis (SEMS-PAGE) gels and transferred onto polyvinylidene fluoride membranes (EMD Millipore, Billerica, MA). The membranes were blocked with 5% skim milk in the presence of the specific primary antibodies, followed by HRP-conjugated secondary antibodies. The protein bands were detected with a chemiluminescence detection kit (GE Healthcare, Little Chalfont, UK).

### Kinase assay

Kinase profiling analysis was performed using human kinases by Reaction Biology Corporation (Malvern, PA). We screened the full panel of kinases (395 kinases). In brief, the kinases were incubated with substrates and necessary cofactors. The reactions were initiated by the addition of orobol in DMSO and ^33^P-ATP (specific activity 10 μCi/μl). After incubation for 120 min at room temperature, the reactions were spotted onto P81 ion exchange paper (GE Healthcare) and washed extensively in 0.75% phosphoric acid. Kinase activity results were expressed as the percent remaining kinase activity in the test samples compared to those of the vehicle (DMSO) reactions. IC_50_ values and curve fits were obtained using Prism (GraphPad Software, La Jolla, CA).

### Pull-down assay

Sepharose 4B freeze-dried powder (0.3 g; GE Healthcare) was activated in 1 mM HCl and suspended in orobol (2 mg) coupled solution (0.1 M NaHCO3 and 0.5 M NaCl). Following overnight rotation at 4 °C, the mixture was transferred to 0.1 M Tris-HCl buffer (pH 8.0) and again further rotated at 4 °C overnight. The mixture was washed three times with 0.1 M acetate buffer (pH 4.0) and 0.1 M Tris-HCl + 0.5 M NaCl buffer (pH 8.0), respectively, and suspended in PBS. The pull down assay was performed as previously described. The active protein CK1ε (SignalChem, Richmond, Canada) was incubated overnight with either sepharose 4B alone or orobol-sepharose 4B beads in reaction buffer [50 mmol/L Tris-HCl (pH 7.5), 5 mmol/L EDTA, 150 mmol/L NaCl, 1 mmol/L DTT, 0.01% NP40, 0.02 mmol/L phenylmethylsulfonyl fluoride]. After incubation at 4 °C, the beads were washed in washing buffer [50 mmol/L Tris-HCl (pH 7.5), 5 mmol/L EDTA, 150 mmol/L NaCl, 1 mmol/L DTT, 0.01% NP40, 0.02 mmol/L phenylmethylsulfonyl fluoride] and proteins bound to the beads were analyzed by immunoblotting.

### Molecular modeling

Insight II (Accelrys Inc, San Diego, USA) was used for the docking study and structure analysis using the coordinates of CK1ε in complex with PF4800567 (PDB accession code 4HNI).

### Animal study

All experimental protocols were approved by the Institutional Animal Care and Use Committee of Seoul National University, Korea (Case Number: SNU-150508-9). All experiments were performed in accordance with relevant guidelines and regulations. Male C57BL/6J mice (5-week-old) were purchased from Japan SLC (Hamamatsu, Shizuoka, Japan). The normal diet (ND) was purchased from Zeigler (Gardners, PA) and high-fat diet (HFD) was purchased from Research Diets (New Brunswick, NJ). Mice were housed in climate-controlled quarters AND a 12-h light–dark cycle. After 1 week of acclimation, mice were divided into three different dietary groups (n = 10 each group): ND, HFD, and a HFD + 10 mg/kg body weight (BW)/day of orobol. Diets were provided in the form of pellets for 23 weeks. Orobol was dissolved in 1% DMSO and 99% PEG200 and administered intragastrically every day. The ND and HFD groups received vehicle (1% DMSO and 99% PEG200). Body weight and food intake were monitored on a weekly basis.

### Statistical analysis

For the *in vitro* study, data were expressed as means ± standard error of the mean (SEM) and the differences between undifferentiated control and the MDI control group were compared using Student’s t-test. To compare the difference between the MDI treated groups, one-way ANOVA followed by Duncan’s statistical range test was used. P values of less than 0.05 were considered as statistically significant. The data was statistically analyzed with IBM SPSS Statistics ver. 22.0 (Armonk, NY).

## Supplementary information

Supplementary information
