# Supplementary material for: Orobol, an Enzyme-Convertible Product of Genistein, exerts Anti-Obesity Effects by Targeting Casein Kinase 1 Epsilon
Source: Sci Rep. 2019 Jun 20;9:8942. doi: 10.1038/s41598-019-43950-9 (PMC6586855; doi:10.1038/s41598-019-43950-9)

Supplementary information

Orobol, an Enzyme-Convertible Product of Genistein, exerts Anti-Obesity Effects by Targeting Casein Kinase 1 Epsilon

Hee Yang[1,2,†], Sang-Hyuk Lee[3,†], Hae Ji[1,2, †], Jong-Eun Kim[4,†], Ra Yoo[1,2], Jong Hun Kim[1,5], Sujin Suk[1,2], Chul Sung Huh[6], Jung Han Yoon Park[2], Yong-Seok Heo[7], Han-Seoung Shin[8], Byung-Gee Kim[9,*], Ki Won Lee[1,2,10,*]

[1]Department of Agricultural Biotechnology and Research Institute of Agriculture and Life Sciences, Seoul National University, Seoul 08826, Republic of Korea

[2]Advanced Institutes of Convergence Technology, Seoul National University, Suwon 16229, Republic of Korea

[3]Interdisciplinary Program for Biochemical Engineering and Biotechnology, Seoul National University, Seoul 08826, Republic of Korea

[4]Research Institute of Biotechnology and Medical Converged Science, Dongguk University, Goyang 10326, Republic of Korea

[5]Department of Food Science and Biotechnology, Sungshin University, Seoul 01133, Republic of Korea

[6]Graduate School of International Agricultural Technology, Seoul National University, Pyeongchang 25354, Gangwon-do, Republic of Korea

[7]Department of Chemistry, Konkuk University, Seoul 05030, Republic of Korea

[8]Department of Food Science and Biotechnology and Food and Biosafety Research Center, Dongguk University-Seoul, Ilsandong-gu, Gyeonggi-do 10326 Republic of Korea

[9]School of Chemical and Biological Engineering, Seoul National University, Seoul 08826, Republic of Korea

[10]BOBSNU Co., Ltd., Suwon 16229, Gyeonggi-do, Republic of Korea

[†]These authors contributed equally to this work.

***Address correspondence to:**

Ki Won Lee, Major in Biomodulation, Department of Agricultural Biotechnology and Research Institute for

Agriculture and Life Sciences, Seoul National University, Seoul 08826, Republic of Korea. Tel.: +82-2-880-4662; Fax: +82-2-873-5095; E-mail: kiwon@snu.ac.kr

Byung-Gee Kim, School of Chemical and Biological Engineering, Seoul National University, Seoul National University, Seoul 08826, Republic of Korea Tel: +82-2-880-6896; Fax: +82-2-882-4158; E-mail: byungkim@snu.ac.kr

Supplement Figure 1. Effects of Orobol blocked the G1 phase during mitotic clonal expansion. Cells were harvested at the indicated time after the initiation of differentiation with or without Orobol treatment and stained with PI solution for flow cytometer cell cycle analysis. The histogram results indicate cell cycle distribution in G1, S or G2/M phases. Data were represented as mean ± SEM values of at least three independent experiments. To compare the differences among the groups, one-way ANOVA was used with Duncan's multiple range tests. Different letters indicated statistically significant differences at $p < 0.05$.

Supplement Figure 2. Effects of CK1ε inhibitor on MDI-induced adipogenesis. (A) After cell differentiation, 3T3-L1 adipocytes were stained with Oil red O and images were captured. (B) Quantification of intracellular lipid content. (C) Orobol does not exhibit cytotoxicity in 3T3-L1 preadipocytes up to 40 μM. Data are represented as means ± SEM from at least three independent experiments. The sharps (##) indicate a significant difference between the control group and the group treated with the MDI cocktail alone ($p < 0.01$). Means with different letters (a–d) within a graph are significantly different between groups treated with MDI alone and those treated with MDI plus PF5006739

Supplement Figure 3. Effects of orobol on MDI-induced signaling in 3T3-L1 preadipocytes.

Western blot analysis for Akt, mTor and $p70^{S6K}$ in MDI-induced signaling in 3T3-L1 preadipocytes. The data are representative of three independent experiments that gave similar results.

Supplement Figure 4. Effects of orobol on the liver weight in HFD-fed mice. Orobol treatment for 23 weeks decreased the weight of liver tissue in HFD-fed mice. Values are expressed as means ± SEM. The sharp (#) indicates a significant difference between the control group and the group treated with HFD ($p < 0.05$). The means marked with '*' indicate significantly different compared to the group treated with HFD ($p < 0.05$)

Supplement Fig.5. These are uncropped blot images corresponding to data show in Figure 3.

Supplement Fig.6. These are uncropped blot images corresponding to data show in Figure 4A.

Supplement Fig.7. These are uncropped blot images corresponding to data show in Figure 4B.

Supplement Table 1. Inhibition of kinase activity by 20 μM orobol

Supplement Figure 1

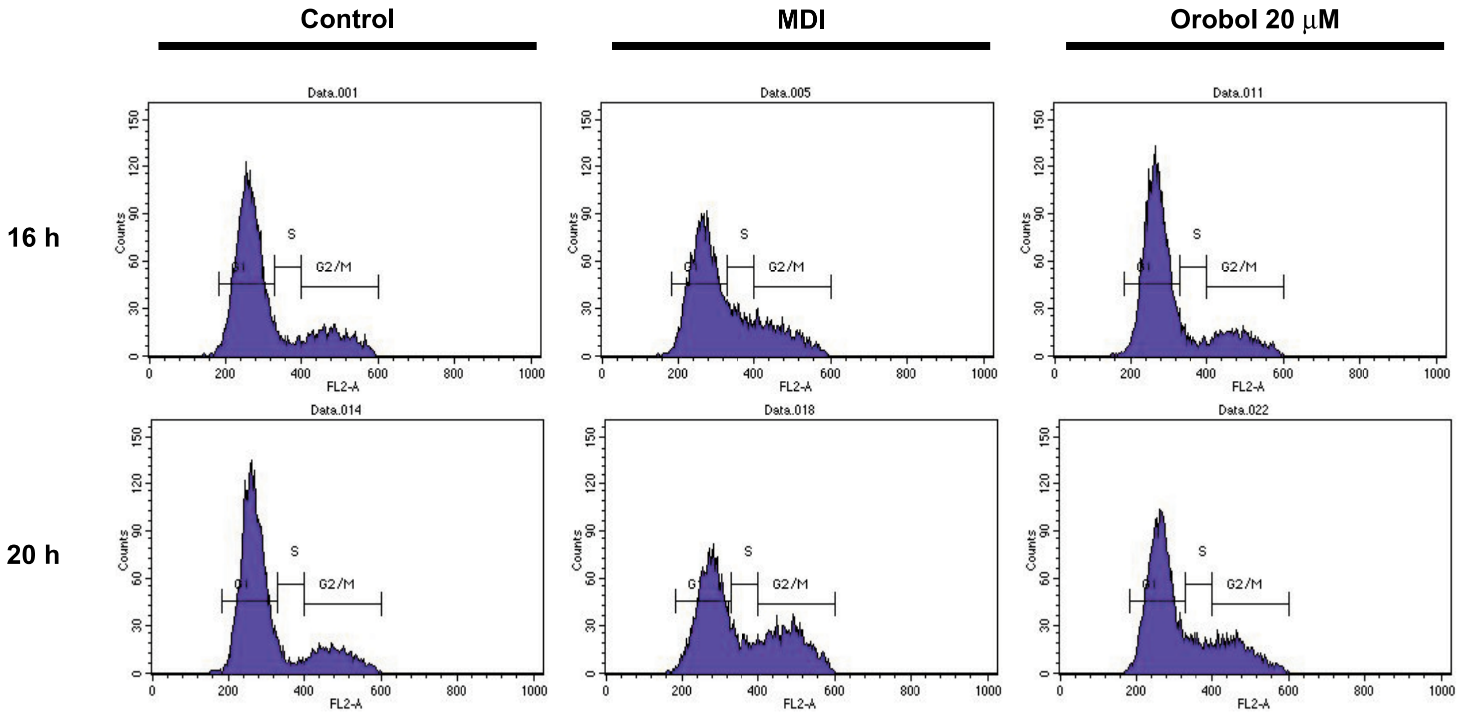

Supplement Figure 2

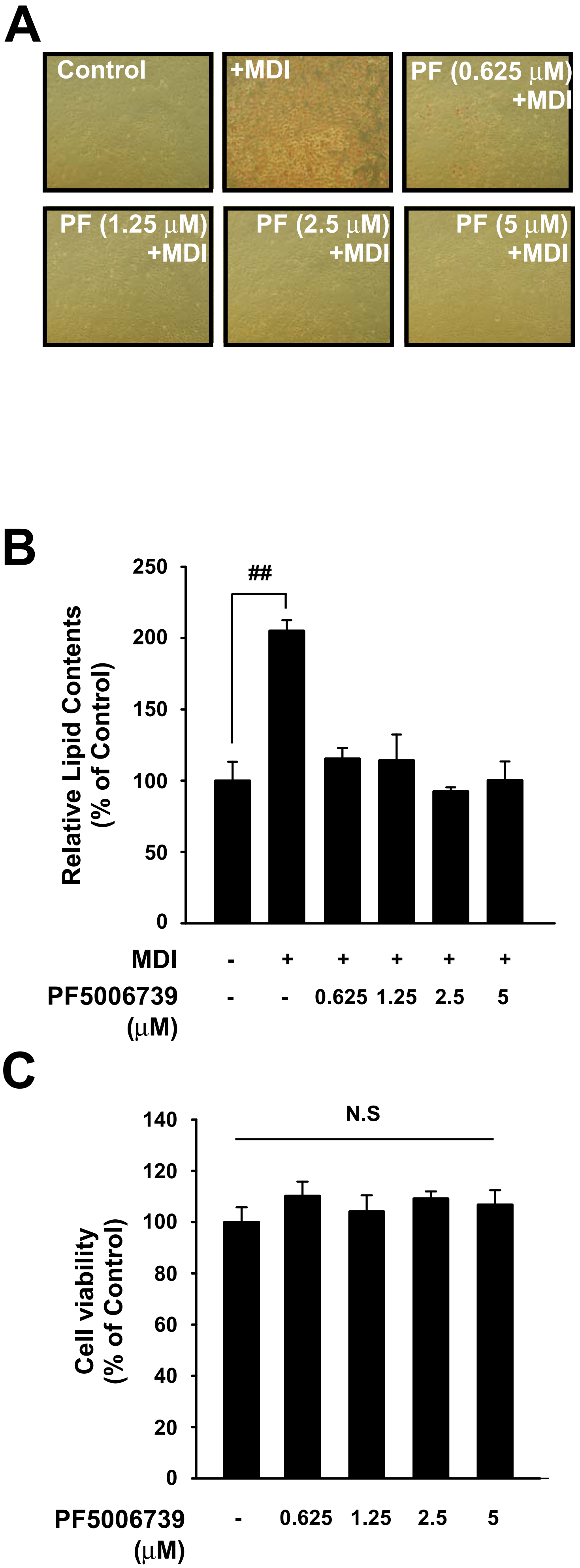

Supplement Figure 3

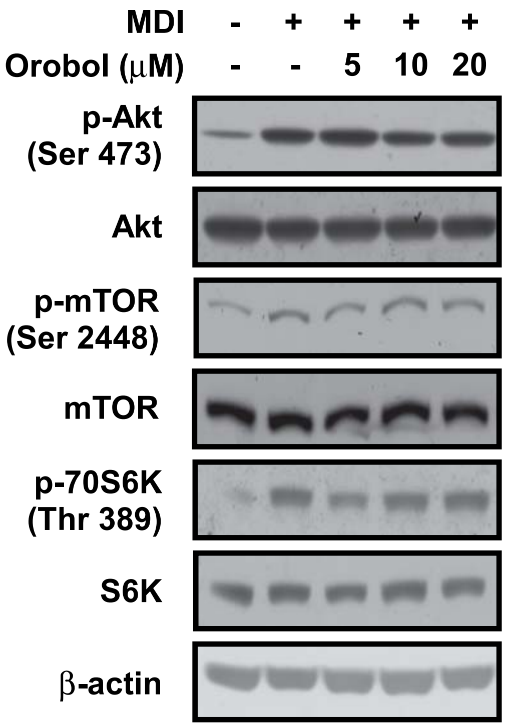

Supplement Figure 4

A

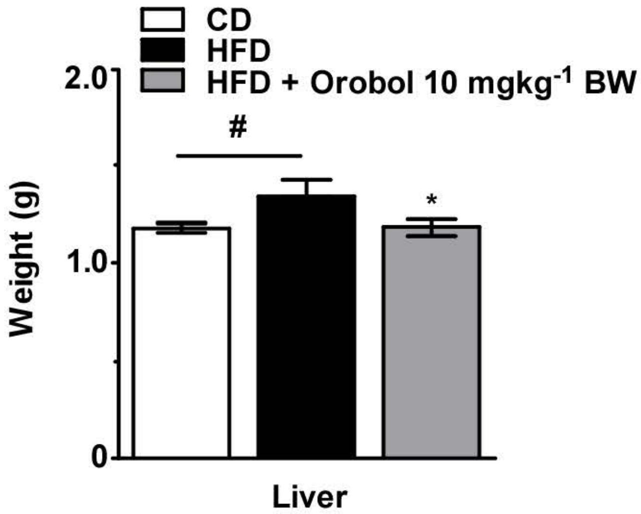

Supplement Figure 5

Figure 3B

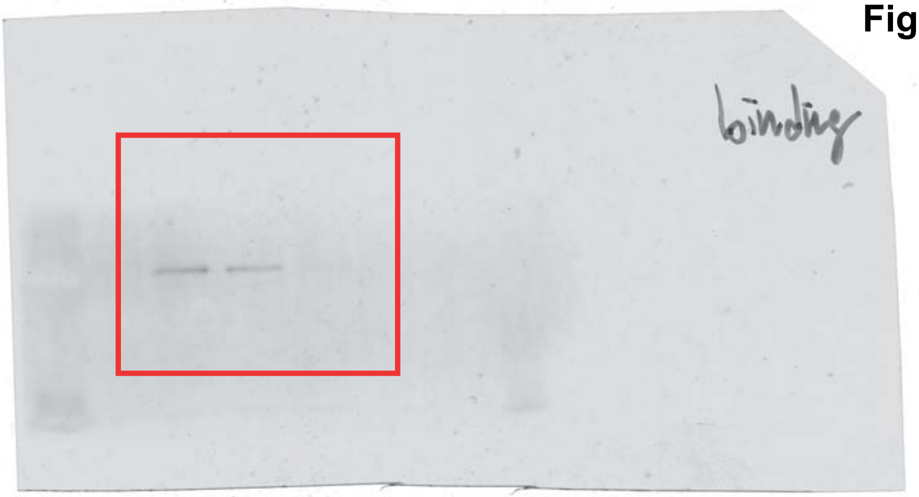

Figure 3B

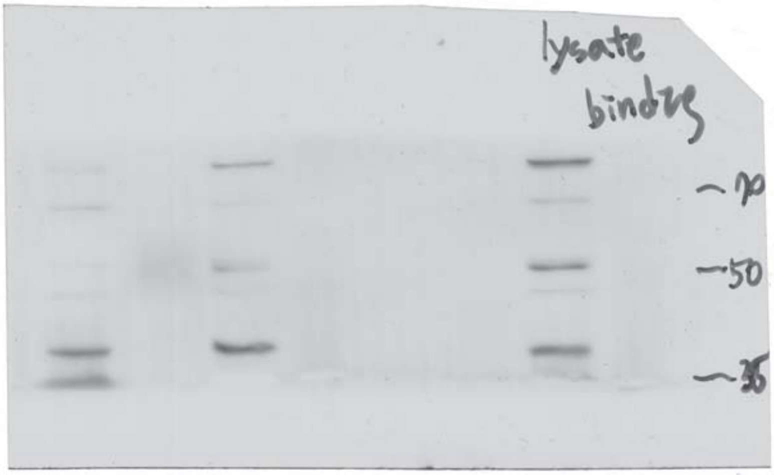

Figure 3B

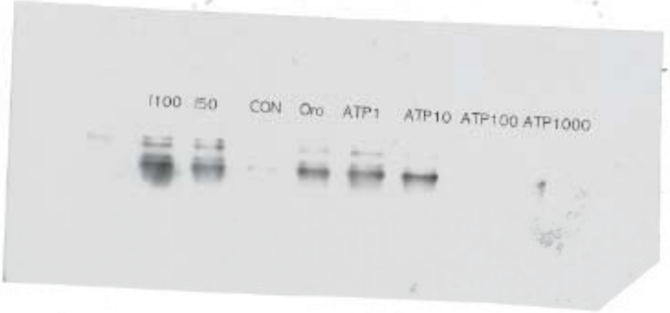

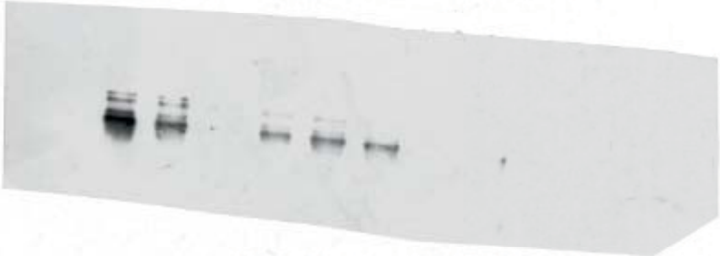

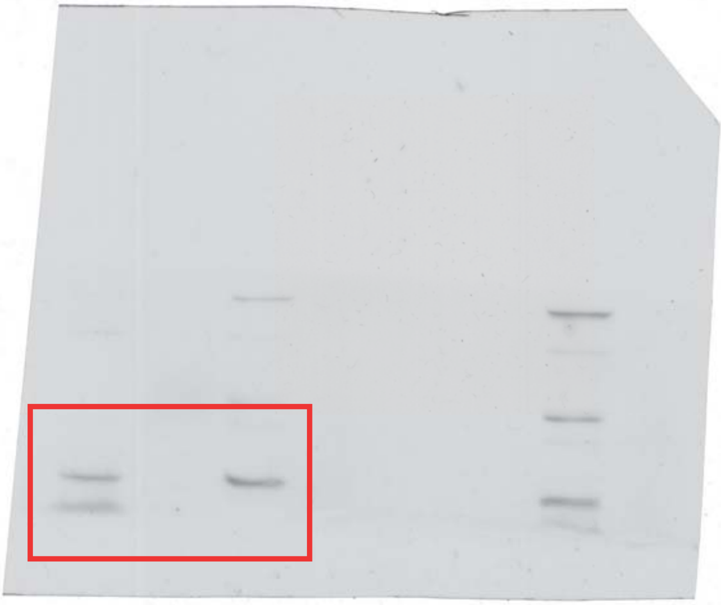

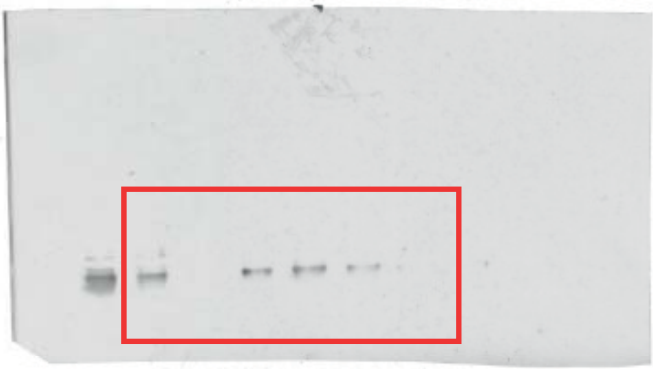

Supplement Figure 6

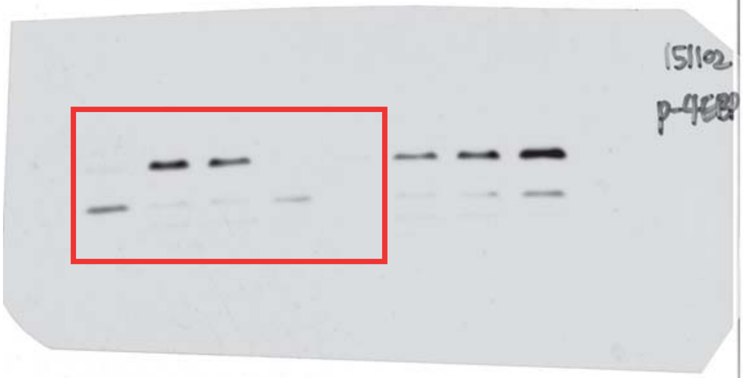

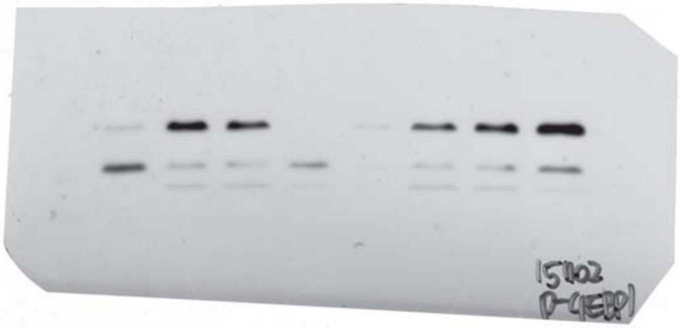

1.Control
2.MDI
3.Orobol 5 +MDI
4.Orobol 10 +MDI
5.Orobol 20 +MDI

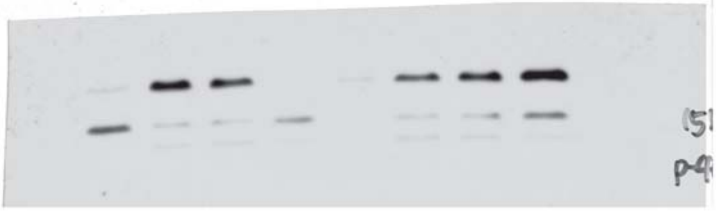

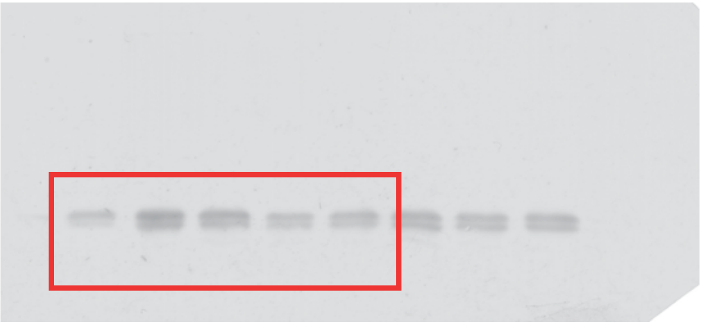

1.Control
2.MDI
3.Orobol 5 +MDI
4.Orobol 10 +MDI
5.Orobol 20 +MDI

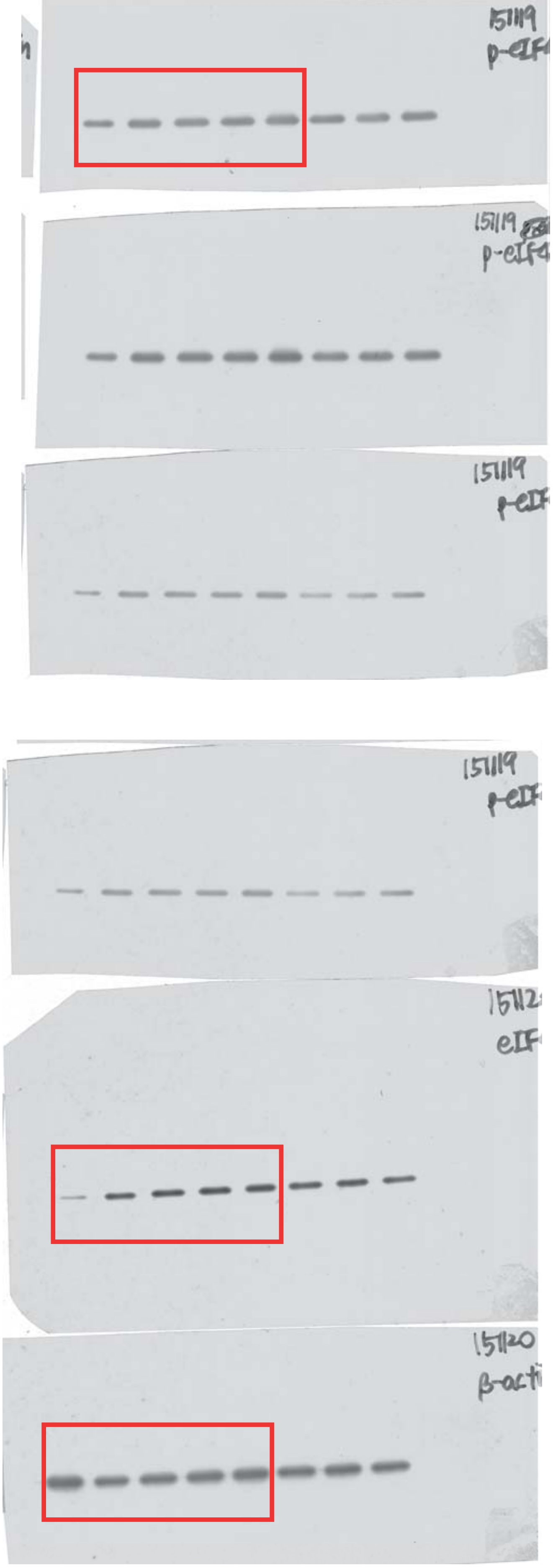

Supplement Figure 7

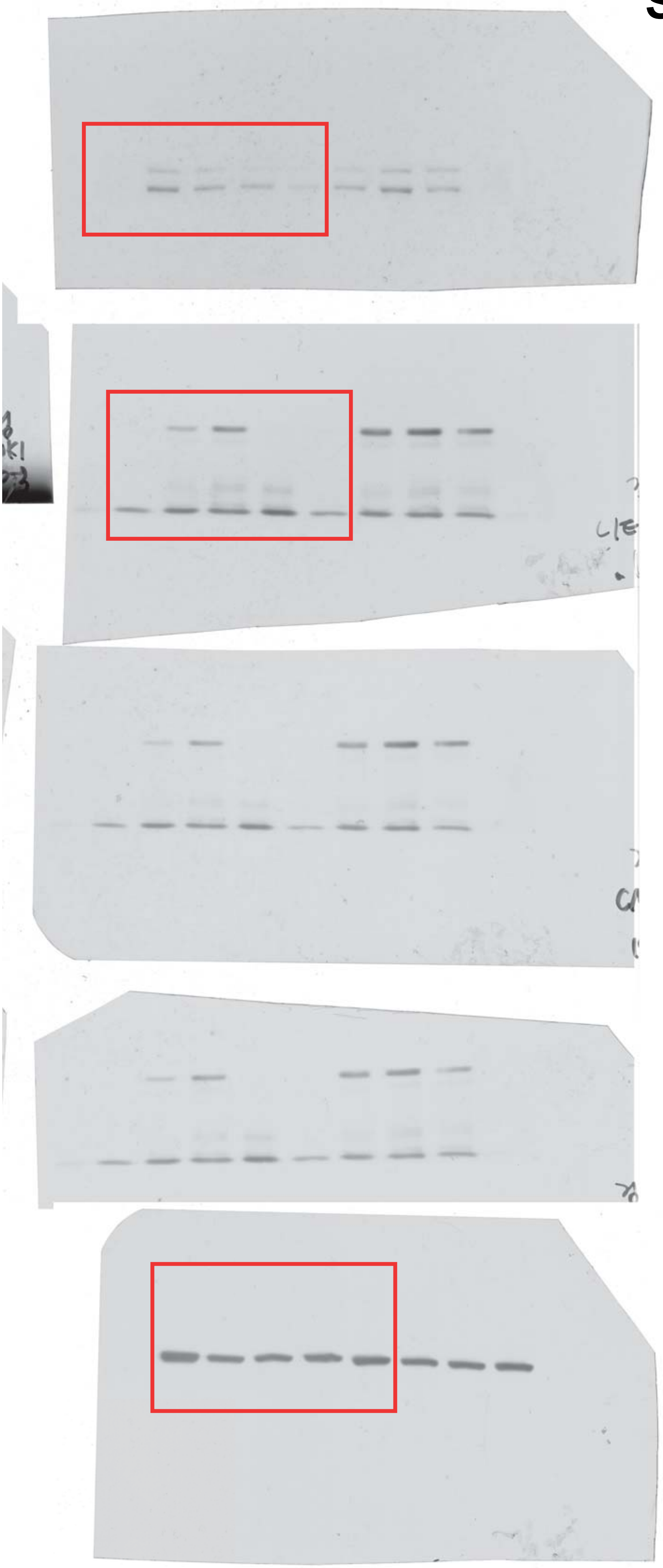

Supplement Figure 8

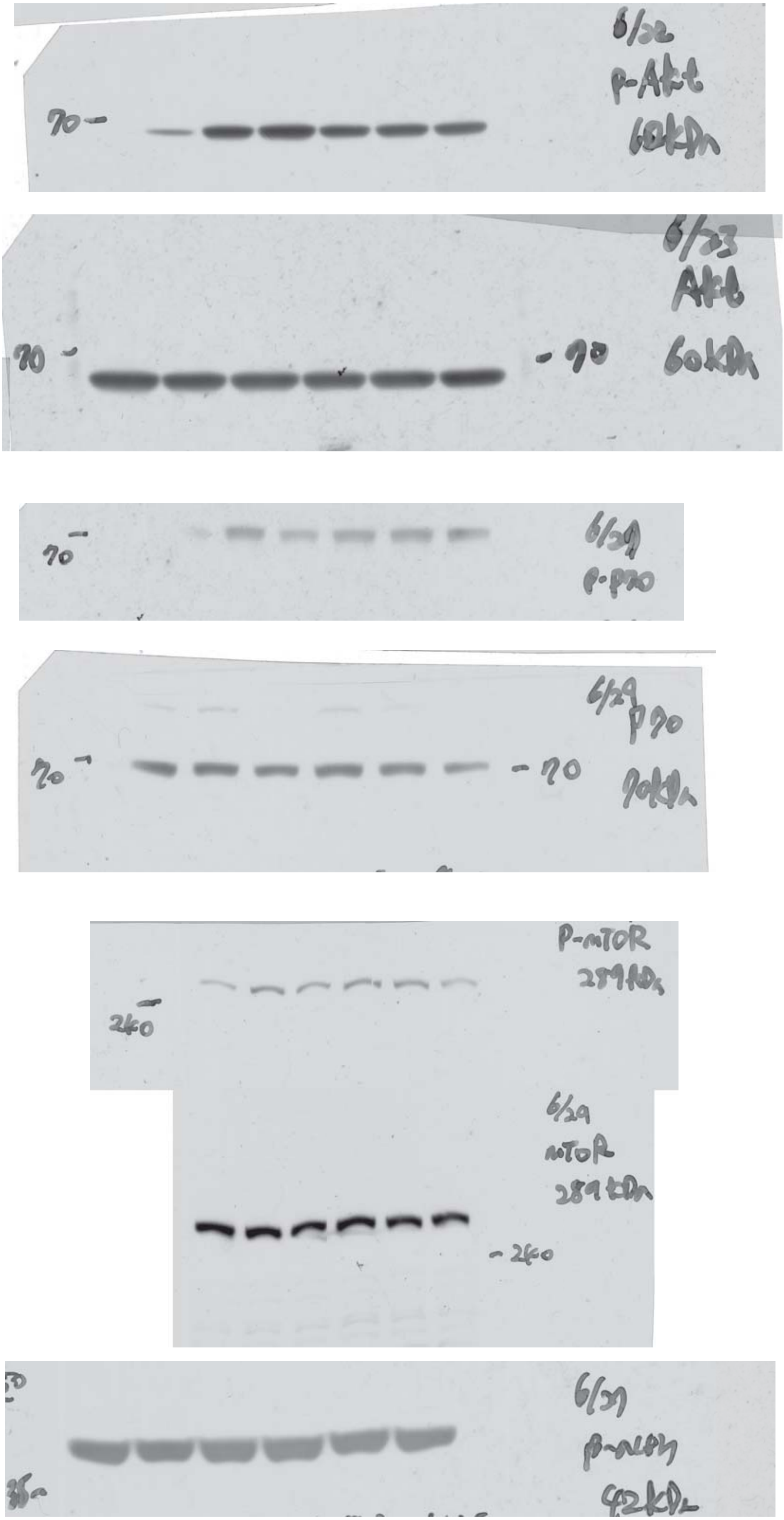

Supplement: Supplementary file 1 — Supplementary information [file 41598_2019_43950_MOESM1_ESM.pdf]
